# Optimum Extraction, Characterization, and Antioxidant Activities of Polysaccharides from Flowers of* Dendrobium devonianum*

**DOI:** 10.1155/2018/3013497

**Published:** 2018-02-08

**Authors:** Donghui Wang, Bei Fan, Yan Wang, Lijing Zhang, Fengzhong Wang

**Affiliations:** ^1^Institute of Food Science and Technology, Chinese Academy of Agricultural Sciences, Beijing 100193, China; ^2^Laboratory of Quality & Safety Risk Assessment on Agro-Products Processing (Beijing), Ministry of Agriculture, Beijing, China

## Abstract

Response surface methodology (RSM) was employed to optimize the conditions for the ultrasonic-assisted extraction (UAE) of polysaccharides from the flowers of* Dendrobium devonianum*. The optimal conditions for the maximum yields of DDFPs are as follows: an extraction temperature of 63.13°C, an extraction time of 53.10 min, and a water-to-raw material ratio of 22.11 mL/g. Furthermore, three fractions (DDFPs30, DDFPs50, and DDFPs70) were prepared from* Dendrobium devonianum* flowers polysaccharides (DDFPs) by the stepwise ethanol precipitation method. The DDFPs50 exhibited the highest antioxidant activity compared to the other fractions. The molecular weight, polydispersity, and conformation of these fractions were also characterized. In particular, the monosaccharide composition analysis of the DDFPs indicates that mannose and glucose are the primary components, similar to those of the* D. officinale* plant. This study provides a rapid extraction technology and essential information for the production of DDFPs, which could be potentially used as healthcare food.

## 1. Introduction

The genus* Dendrobium* belongs to the tribe Dendrobieae, the second largest genus in the family Orchidaceae with approximately 800–1500 species [1]. The* Dendrobium officinale* Kimura et Migo* (D. officinale)* plant, which is an important traditional Chinese herbal medicine, was officially included in the Chinese Pharmacopoeia (2005). The stems and leaves of* D. officinale* exhibit medicinal properties [2–4]. For example, they have been used to cure diabetes, obesity, and rheumatoid arthritis [5, 6]. However, because of overexploitation, the valuable* D. officinale* plant has been on the verge of extinction. Consequently, this herb is in high demand and has a high market value in China. In contrast to* D. officinale* species,* Dendrobium devonianum (D. devonianum)* has been mass-produced in China by tissue cultivation. The local people have the habit of drinking the extracts of the* D. devonianum* flowers (DDFs) as a tea beverage for health benefits. Several studies have demonstrated that polysaccharides are the major bioactive compounds in the* Dendrobium* species [6, 7]. Therefore, there is potential for the commercialization of the polysaccharides from DDFs.

Conventionally, hot-water-infusion technology has been successfully used for polysaccharide extraction [8]. Ultrasonic-assisted extraction (UAE) employs ultrasonic waves to effectively accelerate the release of the target compounds into the extraction solvent. Compared to the conventional extraction techniques, UAE has attracted much interest because of its inherent advantages such as simplified manipulation, significant reduction in energy consumption, lower temperature, and higher efficiency [9]. Response surface methodology (RSM) is a statistical technique for developing, improving, and optimizing processes. The main advantage of RSM is that it is an effective and accurate tool to evaluate multiple variables and their interactions [8].

However, thus far, there are limited studies on the optimization of the extraction process of DDF polysaccharides (DDFPs) as well as their characterization and antioxidant activities. In this study, both UAE and RSM were used to optimize the extraction conditions of DDFPs based on a single-factor preliminary experiment. Three factors (temperature, time, and water-to-raw material ratio) affecting the extraction yield of the polysaccharides were investigated. Then, the* in vitro* antioxidant activities of three ethanol precipitation fractions of DDFPs were investigated. Finally, the aforementioned fractions were characterized by high-performance size-exclusion chromatography coupled with multiangle laser light scattering (HPSEC-MALLS) and high-performance anion-exchange chromatography coupled with pulsed amperometric detector (HPAEC-PAD) analysis.

## 2. Experimental

### 2.1. Materials and Chemicals

The flowers of* D*.* devonianum* were harvested in November 2013 in Baoshan city, Yunnan Province, China. The collected flowers were immediately dried at 60°C for 24 h. The samples were ground and sieved using a grinder and passed through a 40-mesh sieve. Standard monosaccharides, 1,3,5-tri(2-pyridyl)-2,4,6-triazine (TPTZ), and 1,1-diphenyl-2-picrylhydrazyl (DPPH) were purchased from Sigma Chemical Co. (Shanghai, China). Deionized water (18 MΩ cm) was obtained from an NW purified water system (Heal Force, Shanghai, China). All other chemical reagents were of analytical grade.

### 2.2. Extraction of Crude Polysaccharides

Extraction was performed according to a previously published method [10]. Each sample (2.0 g) was extracted using a KQ-600DV ultrasonic instrument (Kunshan Ultrasonic Machinery Co., Jiangsu, China) with distilled water as the solvent at designated temperatures from 30 to 80°C, extraction times at 10, 20, 30, 40, 50, 60, 70, and 80 min, respectively, and water-to-raw material at ratios from 10 to 35 mL/g. The extracts of each sample were cooled at room temperature and filtered, and then the polysaccharide concentration of the DDFPs was determined by the phenol–sulfuric acid method [11], with glucose solutions (50, 100, 150, and 200 *μ*g/mL) as the standards.

The polysaccharide extraction yield (*Y*) was calculated as follows:

(1) $$\begin{array}{c} Y\;\left(\;\%\;\right)=\frac{\left(100\times W_{DDFPs}\right)}{W_{sample}}, \end{array}$$

where *W*_DDFPs_ is the weight of the DDFPs and *W*_sample_ is the weight of the sample.

### 2.3. RSM Experimental Design

The conditions for the UAE of polysaccharides from DDFs were optimized by RSM. To investigate these conditions, the three variables for extraction—temperature, extraction time, and water-to-raw material ratio—were denoted as labels *A*, *B*, and *C*, respectively. Using RSM with a Box–Behnken design (BBD), the three independent variables were investigated in terms of the yield of DDFPs (denoted as *Y*), as shown in Table 1. The three independent variables (*A*, *B*, and *C*) were divided into three levels, coded +1, 0, and −1, for high, intermediate, and low values, respectively. The total experimental system was composed of 12 factorial experiments based on the BBD and five repeated tests at the center point.

### 2.4. Preparation of Three DDFP Fractions by the Stepwise Ethanol Precipitation Method

The DDFPs (1.0 g) were dissolved in 100 mL of deionized water. Then, the solution was adjusted to the final ethanol concentration of 30% using 95% ethanol and stored at 4°C overnight. The residue obtained after the precipitation was centrifuged at 10000 ×g and 25°C for 20 min and freeze-dried to obtain a dry fraction, denoted as DDFPs30. Similar cycles were performed to prepare the fractions of DDFPs50 and DDFPs70 by adjusting the final precipitation ethanol concentration to 50% and 70%, respectively.

### 2.5. Measurement of Antioxidant Activities

#### 2.5.1. DPPH Radical-Scavenging Assay

The DPPH radical-scavenging capacity assay was based on a 96-well microplate method [12]. Briefly, 20 *μ*L of three DDFP fractions (DDFPs30, DDFPs50, and DDFPs70) was mixed with 100 *μ*L of a methanolic solution of DPPH (0.2 mM) and left for 30 min at room temperature before the absorbance was recorded at 517 nm using the microplate reader (MD190, Santa Barbara, CA, USA). The antioxidant activities of the radical scavengers in different polysaccharides were investigated. The radical-scavenging activities were calculated as percentages using the following equation:

(2) DPPH  radical-scavenging  activity %=ADPPH−A1−ASADPPH×100%,

where *A*_DPPH_ is the absorbance of DPPH, *A*_1_ is the absorbance of DPPH and the sample extract, and *A*_*S*_ is the absorbance of the sample extract. All the samples were tested in triplicate.

#### 2.5.2. Ferric Reducing Antioxidant Power (FRAP) Assay

The FRAP assay was also conducted according to our previous method using 96-well microplates [12]. The samples (10 *μ*L) were mixed with 300 *μ*L of ferric-TPTZ reagent (prepared by mixing 300 mM acetate buffer, pH 3.6, 10 mM TPTZ in 40 mM HCl, and 20 mM FeCl_3_·7H_2_O in a ratio of 10 : 1 : 1 (v/v/v)). After 30 min of incubation at room temperature, the data were recorded at an absorbance of 593 nm against a blank (the FRAP reagent was previously prepared without the extracts). The data obtained with FeSO_4_·7H_2_O from the calibration curve were calculated using the following equation: average *R*^2^ = 0.9977, *y* = 0.005*x* + 0.0085. The antioxidant activities were expressed as micromoles of FeSO_4_ equivalent per gram dry weight (*μ*mol Fe^2+^E/g DW). All the samples were tested in triplicate.

### 2.6. Characterization of DDFPs

#### 2.6.1. Molecular Weight and Size Distribution Analysis

Molecular weight and molecular size distribution were investigated using an HPSEC system coupled to an integrated detector array: a refractive index detector (Wyatt Technology, Santa Barbara, CA, USA), a UV L-2400 detector (Hitachi High Technologies America, Inc., Schaumburg, Illinois, USA), and a MALLS detector (Wyatt Technology, Santa Barbara, CA, USA). The chromatographic system consisted of an L-2130 pump (Hitachi Scientific Instruments Inc., Columbia, Maryland, USA) and a TOSOH TSKgel G4000PWXL column (300 mm × 7.8 mm i.d., Tokyo, Japan). Each polysaccharide (2 mg mL^−1^) of DDFPs30, DDFPs50, and DDFPs70 was subjected to the HPSEC system. The eluent consisted of a 0.1 mol L^−1^ NaNO_2_ solution and 0.5 g L^−1^ NaN_3_ at a flow rate of 0.5 mL min^−1^ with a run time of 25 min. The weight-average molecular weight (*M*_*w*_), number-average molecular weight (*M*_*n*_), and polydispersity index (*M*_*w*_/*M*_*n*_) were analyzed by the Astra software (version 4.73.04, Wyatt Technology, Santa Barbara, CA, USA).

#### 2.6.2. Monosaccharide Composition Analysis

DDFPs30, DDFPs50, and DDFPs70 (5 mg each) were hydrolyzed in 2 mL of 2 M trifluoroacetic acid at 120°C for 6 h. Excess trifluoroacetic acid was removed by codistillation [13]. The monosaccharide compositions of DDFPs30, DDFPs50, and DDFPs70 were analyzed using a HPAEC-PAD (Dionex Technology, Sunnyvale, CA, USA) after acid hydrolysis. HPAEC was performed using a Dionex ICS-3000 system with a Carbo PAC™ PA10 analytical column (4.0 mm × 250 mm) and a PAC PA10 guard column (4.0 mm × 50 mm). The samples (10 *μ*L) were injected into the column to analyze the monosaccharide and eluted with 9% NaOH and NaAC by decreasing the proportion of NaOH (0–20 min: 9% NaOH; 20–30 min: 9% NaOH + 5% NaAC; 35-36 min: 9% NaOH + 20% NaAC). The flow rate was adjusted to 1 mL min^−1^, and the column temperature was set at 25°C. A standard curve was established with different concentrations (0.2, 0.5, 1, 5, and 10 mg/mL) of mixed standard solutions including glucose (Glu), mannose (Man), galactose (Gal), L-arabinose (Ara), *α*-L-rhamnose (Rha), fructose (Fru), galacturonic acid (Gal acid), and glucuronic acid (Glu acid).

## 3. Results and Discussion

### 3.1. Single-Factor Experiments

The RSM optimization of the UAE conditions was based on the maximum DDFP yield of the sample. All the parameters (*A*–*C*) were investigated by single-factor experiments in a wide range prior to the RSM optimization. This helped to narrow down the ranges of the parameters.

**Figure 1:**
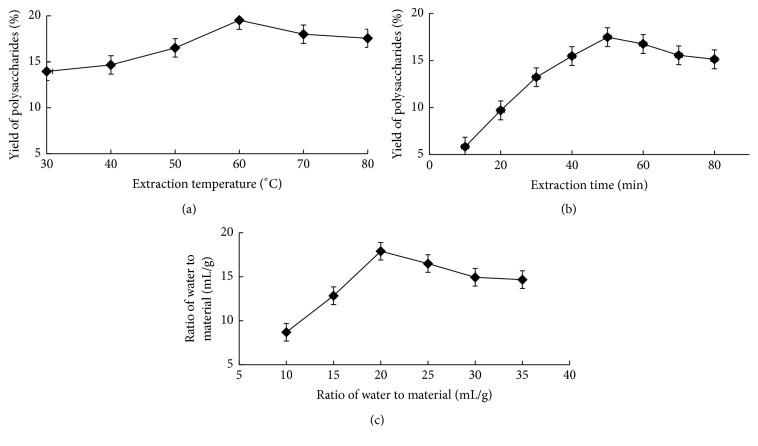
Effects of the extraction temperature (a), extraction time (b), and water-to-material ratio (c) on yield of DDFPs.

The effect of various temperatures (30, 40, 50, 60, 70, and 80°C) on the extraction efficiency of DDFPs was investigated by maintaining the other two factors (extraction time and water-to-raw material ratio) constant at 60 min and 30 mL/g, respectively. As shown in Figure 1(a), the DDFP yield significantly increased with the increase in temperature from 30 to 60°C. The yield was the highest at 60°C and then decreased with increasing temperature. At a higher temperature, the viscosity of the extracts decreased, thus increasing the solubility of the DDFPs, which in turn accelerated the release and dissolution of these compounds. A similar trend has been reported for polysaccharide extraction [14]. To prevent the yield loss and minimize the adverse effects of processing, 70°C was set as the highest temperature in this study. Therefore, the temperature range from 50 to 70°C was used as the optimal condition in the further design of the RSM experiment.

Extraction time is also one of the important variables affecting the extraction efficiency of polysaccharides from natural products [15]. The extraction time was set at 10, 20, 30, 40, 50, 60, 70, and 80 min while maintaining an extraction temperature of 60°C and water-to-raw material ratio at 30 mL/g. As shown in Figure 1(b), the extraction efficiency clearly increased as the extraction time increased from 10 to 50 min. After 50 min, the extraction efficiency slightly decreased with increasing extraction time, with continuous extraction to 80 min. It has also been reported that processing at a long extraction time may lead to the degradation, aggregation, and hydrolysis of polysaccharides [14]. Thus, an extraction time from 40 to 60 min was selected as the optimum condition.

The yields of the DDFPs extracted by different water-to-raw material ratios (10, 15, 20, 25, 30, and 35 mL/g) are shown in Figure 1(c) while maintaining an extraction temperature of 60°C and an extraction time of 60 min. The solvent volume in the studied range played an important role in the UAE extraction of the DDFPs. This may be caused by the fact that less amounts of polysaccharides can be extracted using a small quantity of an extraction solvent (water-to-raw material ratio = 10 mL/g). Increasing the water-to-raw material ratio to 20 mL/g increased the DDFP yield. Then, at the ratio 20 mL/g, all the polysaccharides could be extracted from DDF. Thus, the water-to-raw material ratio was set in the range 15–25 as the optimal condition.

Thus, the RSM experiments were conducted under the following conditions: an extraction temperature of 50–70°C, an extraction time of 40–60 min, and a water-to-raw material ratio of 15–25 mL/g.

### 3.2. Response Surface Model (Statistical Analysis)

**Figure 2:**
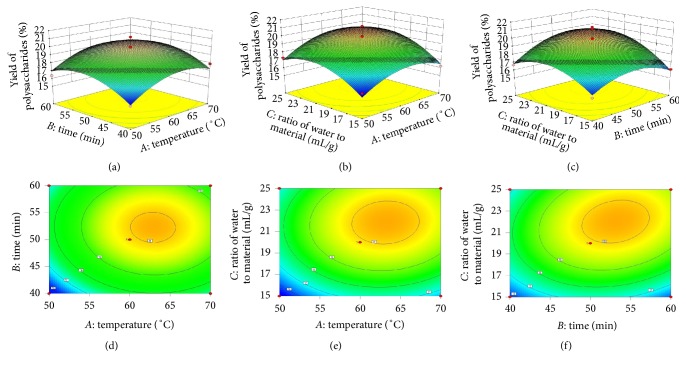
Response surface plots (a, b, and c) and contour plots (d, e, and f) showing the effect of time, temperature, and water-to-material ratio on yield of DDFPs.

A total of 17 runs were designed to evaluate the three independent variables *A*, *B*, and *C* in the BBD, as shown in Table 1. The predicted model sufficiently explained the response. The independent and dependent variables are expressed by the following equation: *Y* = 19.92 + 0.82*A* + 0.66*B* + 1.09*C* − 0.050*AB* + 0.18*AC* + 0.55*BC* − 1.42*A*^2^ − 1.41*B*^2^ − 1.56*C*^2^.  Table 2 shows the model results of the independent variables on the extraction yield evaluated by the analysis of variance. The results indicate that the linear terms of *A*, *B*, and *C* were all significant (*P* < 0.05) to *Y*; in particular, *C* was very significant. Not all of the cross terms (*AB*, *AC*, and *BC*) were significant, and all the quadratic terms (*A*^2^, *B*^2^, and *C*^2^) were very significant to *Y* (*P* < 0.01). The model used to fit the response variable was significant (*P* < 0.05) and adequate to represent the relationships between the response and independent variables. A “lack-of-fit *F*-value” of 0.758064 indicates that the lack-of-fit is not significantly relative to the pure error. There is a 57.31% chance that the lack-of-fit *F*-value is large, possibly because of noise. The total determination coefficient (*R*^2^) and adjusted determination coefficient were 0.9200 and 0.8172, respectively, confirming that the model is reasonable and significant. The effects of the independent variables and their mutual interaction on the extraction yield were observed on the response surface and contour plots, as shown in Figure 2. The optimum conditions for the yield of the DDFPs are as follows: an extraction temperature of 63.13°C, an extraction time of 53.10 min, and a water-to-raw material ratio of 22.11 mL/g. Accordingly, the theoretical highest yield of DDFPs was predicted as 20.3779% by the developed model. Verification experiments were conducted by utilizing the modified conditions of an extraction temperature of 65°C, an extraction time of 55 min, and a water-to-raw material ratio of 22 mL/g for the three replicates. The average yield of the DDFPs was 20.25%. These experimental yields were in good agreement with those predicted by the model.

### 3.3. Evaluation of Antioxidant Activity of Three Fractions from DDFPs

To further select the polysaccharide fraction with the highest antioxidant activity, three fractions (DDFPs30, DDFPs50, and DDFPs70) were prepared by the stepwise ethanol concentration precipitation method. Ethanol precipitation is an effective method for fractionation and purification of water-soluble DDFPs in aqueous solutions. In an aqueous solution, the DDFPs molecule exposes its charged and polar residues on the surface to maximize the contact with water molecules. Compared with other isolation methods for biopolymers such as chromatography and membrane, ethanol precipitation has the advantages of simple equipment and easy operation.

**Figure 3:**
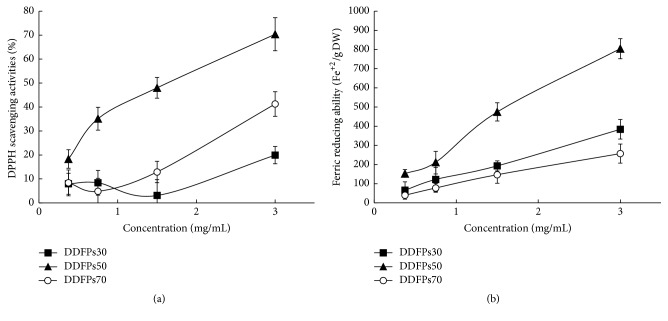
Antioxidant activity of three fractions from DDFPs. (a) Scavenging effect on DPPH radicals. (b) Reducing power evaluation.

As shown in Figure 3(a), all three fractions exhibited scavenging effects, which almost correlated positively with increasing concentrations. The scavenging effect of three fractions increased in the order of DDFPs50 > DDFPs70 > DDFPs30, and their scavenging activities were 70.37 ± 6.89%, 41.26 ± 5.13%, and 19.93 ± 3.61%, respectively, at a concentration of 3 mg/mL.

The FRAP assay is a simple, reproducible, rapid, and inexpensive method to measure the reductive ability of an antiradical and is evaluated by the transformation of ferric ion (Fe^3+^) to ferrous ion (Fe^2+^), as a measure of the total antioxidant capacity [16]. The concentration of three fractions increased the FRAP value in a dose-dependent manner (Figure 3(b)). Moreover, DDFPs50 also exhibited higher antioxidant activity (804.56 ± 52.70 *μ*mol Fe^2+^E/g DW) than DDFPs30 (383.89 ± 51.03 *μ*mol Fe^2+^E/g DW) and DDFPs70 (257.11 ± 50.02 *μ*mol Fe^2+^E/g DW).

### 3.4. Characterization of Three Fractions from DDFPs

#### 3.4.1. Molecular Weight, Polydispersity, and Conformation

To better explore the structural difference between three fractions (DDFPs30, DDFPs50, and DDFPs70) from DDFPs, HPSEC-MALLS was used to determine the molecular weight (*M*_*w*_), size distribution, root mean square (RMS), and slope exponent of a conformation plot (Table 3). The results indicate that *M*_*w*_ of DDFPs30, DDFPs50, and DDFPs70 ranged from 3.78 × 10^5^ to 5.63 × 10^5^. DDFPs70 had the highest *M*_*w*_, followed by DDFPs30 and DDFPs50. The polydispersity indices of these fractions were 1.28, 1.33, and 1.02, respectively. The slope exponent of all these fractions ranged from 0.32 to 0.35. Theoretically, a slope of 0.33 indicates a sphere and slopes of 0.5–0.6 and close to 1.0 indicate a random coil and rigid rod, respectively [17]. The results indicate that all these fractions have more rod-like structures.

#### 3.4.2. Monosaccharide Composition Analysis

**Figure 4:**
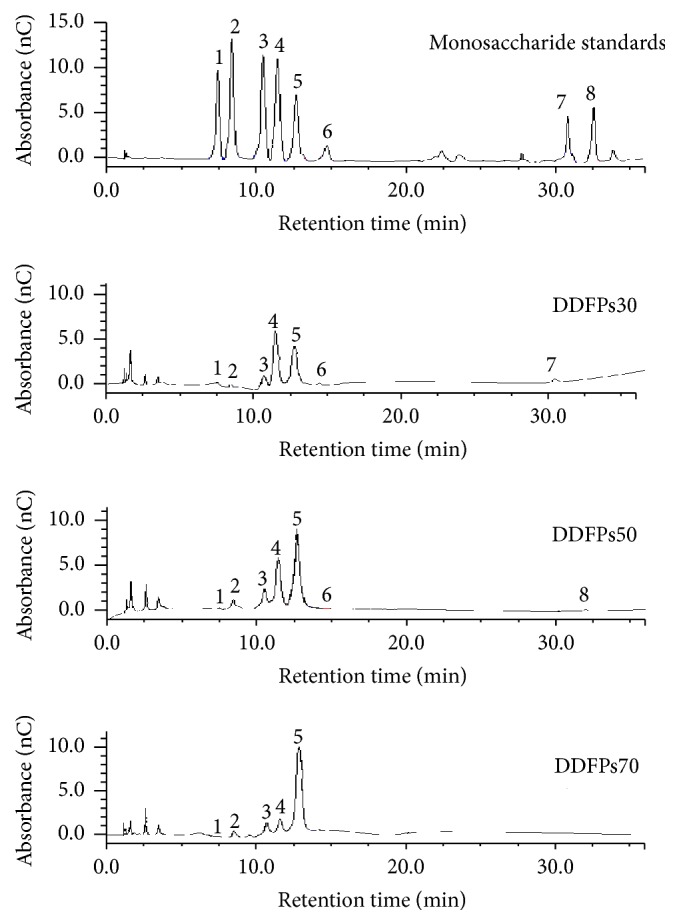
HPAEC-PAD analysis profiles of monosaccharide composition in three fractions from DDFPs. Each peak was separated as described as 1: *α*-L-rhamnose, 2: L-arabinose, 3: galactose, 4: glucose, 5: mannose, 6: fructose, 7: galacturonic acid, and 8: glucuronic acid.

The HPAEC-PAD analysis profiles and data of the monosaccharide compositions of the three polysaccharides from the flowers of* D. devonianum* are shown in Figure 4. The molar ratio of the monosaccharide compositions in DDFPs30 was as follows: Man : Glu : Gal : Rha : Ara : Fru : Gal acid = 4.77 : 5.18 : 1.00 : 0.46 : 0.64 : 0.50 : 1.39. DDFPs50 was composed of Man : Glu : Gal : Rha : Ara : Fru : Glu acid in a molar ratio of 8.45 : 2.93 : 1.00 : 0.06 : 0.37 : 0.04 : 0.2. DDFPs70 was composed of Man : Glu : Gal : Rha : Ara in a molar ratio of 9.00 : 1.43 : 1.00 : 0.28 : 0.59. The purified* O*-acetyl-glucomannan in* D. officinale* herbal materials was mainly composed of mannose and glucose in a molar ratio of 6.9 : 1 [5]. Our result showed that the DDFPs had similar monosaccharide composition.

## 4. Conclusions

In this study, RSM was applied for the first time to determine the optimal conditions for the extraction of DDFPs. The optimum conditions for the yield of DDFPs are as follows: an extraction temperature of 63.13°C, an extraction time of 53.10 min, and a water-to-raw material ratio of 22.11 mL/g. Furthermore, under the optimized conditions, the yield obtained from the verification experiments (20.25%) agreed well with the theoretical yield (20.38%), indicating that the regression model is efficient and successful for the extraction of DDFPs from DDFs. To further select the fraction with higher antioxidant activity, the stepwise ethanol precipitation method was used to separate the fractions from DDFPs as DDFPs30, DDFPs50, and DDFPs70. DDFPs50 exhibited the highest antioxidant activity in both the DPPH and FRAP assays. *M*_*w*_ of three fractions ranged from 3.78 × 10^5^ to 5.63 × 10^5^. The polydispersity indices of these fractions were 1.28, 1.33, and 1.02, respectively. The slope of the conformation plot indicated that all three fractions had rod-shaped structures. Interestingly, the results showed that the DDFPs had similar monosaccharide composition as the* D. officinale* plant, mainly composed of Man and Glu. Further research is required to study the accurate structure by methylation analysis and two-dimensional NMR spectroscopy. This study provides a rapid extraction technology for the production of DDFPs, which can be potentially used as a new type of antioxidant or a healthcare food.

## Figures and Tables

**Table 1:** Response surface analysis program and results for the yield polysaccharides from *D. devonianum* flowers.

Run	Temperature *A*: temperature (°C)	Time *B*: extraction time (min)	Factor 3 *C*: water-to-material ratio	Yield of polysaccharides *Y*: (%)
1	50 (−1)	50 (0)	25 (1)	17.30
2	60 (0)	60 (1)	15 (−1)	16.11
3	60 (0)	40 (−1)	25 (1)	16.68
4	60 (0)	50 (0)	20 (0)	21.22
5	70 (1)	50 (0)	15 (−1)	16.22
6	50 (−1)	50 (0)	15 (−1)	15.66
7	60 (0)	40 (−1)	15 (−1)	15.41
8	60 (0)	60 (1)	25 (1)	19.57
9	60 (0)	50 (0)	20 (0)	19.4
10	50 (−1)	60 (1)	20 (0)	16.37
11	50 (−1)	40 (−1)	20 (0)	15.42
12	60 (0)	50 (0)	20 (0)	19.02
13	60 (0)	50 (0)	20 (0)	20.00
14	60 (0)	50 (0)	20 (0)	19.94
15	70 (1)	60 (1)	20 (0)	18.65
16	70 (1)	40 (−1)	20 (0)	17.90
17	70 (1)	50 (0)	25 (1)	18.58

**Table 2:** ANOVA for response surface quadratic model: analysis of variance table (partial sum of squares). *A*: temperature (°C), *B*: extraction time (min), and *C*: water-to-material ratio.

Source	Sum of squares	DF	Mean square	*F* value	*P* value	Significant
Model	50.10	9	5.566395	8.944987	0.0043	*∗*
*A*	5.45	1	5.445	8.74991	0.0212	*∗*
*B*	3.50	1	3.498013	5.621174	0.0495	*∗*
*C*	9.53	1	9.526613	15.30891	0.0058	*∗∗*
*A* ^2^	0.01	1	0.01	0.01607	0.9027	
*B* ^2^	0.1296	1	0.1296	0.208262	0.6620	
*C* ^2^	1.199025	1	1.199025	1.926788	0.2077	
*AB*	8.451287	1	8.451287	13.5809	0.0078	*∗∗*
*AC*	8.421487	1	8.421487	13.53301	0.0079	*∗∗*
*BC*	10.23689	1	10.23689	16.45029	0.0048	*∗∗*
Residual	4.356045	7	0.622292			
Lack-of-fit	1.578925	3	0.526308	0.758064	0.5731	
Pure error	2.77712	4	0.69428			
Cor. total	54.4536	16				

^*∗*^ *P* < 0.05 and ^*∗∗*^*P* < 0.01.

**Table 3:** The physical properties of polysaccharides from *D. devonianum* flowers (*n* = 3).

Fractions	*M*_*w*_/*M*_*n*_ (10^5^ g/mol)	Polydispersity (*M*_*w*_/*M*_*n*_)	Slope
DDFPs30	5.41 ± 0.23	1.28 ± 0.15	0.33 ± 0.02
DDFPs50	3.78 ± 0.12	1.33 ± 0.09	0.35 ± 0.03
DDFPs70	5.63 ± 0.14	1.02 ± 0.05	0.32 ± 0.02
